# Investigating the shared genetic architecture between dry eye disease and multisite chronic pain

**DOI:** 10.3389/fmed.2026.1918770

**Published:** 2026-09-21

**Authors:** Gangyi Li, Chengzhang Luo, Shuangle Li

**Affiliations:** 1 Department of Ophthalmology, Zigong First People's Hospital, Zigong, Sichuan, China; 2 Department of Optometry and Ophthalmology, North Sichuan Medical College, Nanchong, Sichuan, China

**Keywords:** cross-trait meta-analysis, dry eye, genome-wide association studies (GWAS), multisite chronic pain (MCP), shared genetic architecture

## Abstract

**Background:**

The epidemiological link between dry eye disease (DED) and chronic pain is established, yet a shared genetic etiology remains uncertain.

**Methods:**

Utilizing GWAS summary data, we applied linkage disequilibrium score regression (LDSC) to determine genetic correlation between DED and multisite chronic pain (MCP). We investigated SNP enrichment across tissues using stratified LD score regression (S-LDSC) and multimarker analysis of genome annotation (MAGMA). Common risk SNPs were identified via Multi-Trait Analysis of GWAS (MTAG) and Cross-Phenotype Association (CPASSOC), followed by pleiotropic analysis under the composite null hypothesis (PLACO) and colocalization. Multi-trait colocalization (Hyprcoloc) integrating brain imaging-derived phenotypes (IDPs) was applied to explore neuroimaging mediators. We further investigated risk gene expression profiles using Summary-data-based Mendelian randomization (SMR) to explore functional genes.

**Results:**

We found significant genetic correlation between DED and MCP, highlighting shared risk SNPs (rs13107325, rs7105462, rs7959961). Hyprcoloc revealed rs13107325 as a central hub with three-way colocalization across 95 IDPs. Partitioned analysis identified 11 functional elements associated with both phenotypes. Tissue enrichment converged on brain regions, notably the anterior cingulate cortex and cerebellar hemispheres. SMR identified 21 shared functional genes.

**Conclusion:**

This study suggests genetic correlation and common risk genes between DED and MCP, advancing understanding of their pathogenesis and assisting in identifying therapeutic targets.

## Introduction

Chronic pain, defined as pain persisting or recurring for more than 3 months, affects approximately 30% of the global population and represents a major reason for seeking medical care and one of the leading causes of disability worldwide (1, 2). In the United States, the economic burden of chronic pain is substantial, with estimated annual costs ranging from $560 billion to $635 billion (3). Chronic pain frequently leads to mental disorders and physical comorbidities, and the relationship between chronic pain and dry eye disease (DED) has attracted increasing attention. Previous reports have indicated that patients with chronic pain may be more susceptible to DED, presenting with both signs and symptoms (4, 5). Further studies have demonstrated that chronic pain alters corneal nerve architecture (6–8). The underlying mechanisms may include peripheral sensitization-induced hypersensitivity of nociceptors and abnormal central sensory processing, all of which may contribute to the onset and progression of DED. Given that the global prevalence of DED ranges from 5% to 50% (9, 10), it has become a significant public health concern affecting quality of life; therefore, understanding the shared genetic architecture between chronic pain and DED is critical and essential for the management of DED.

Chronic pain and DED both have substantial genetic underpinnings. Studies have shown that the heritability of DED is estimated at 41% (11), while the overall heritability of chronic pain ranges from 35% to 50% (12). To date, few studies have explored the connection between DED and chronic pain at the genetic level, and it remains unclear whether and how their genetic architectures overlap. Based on the established association between DED and chronic pain in epidemiological studies, we hypothesized that DED and chronic pain may share a common genetic architecture. Based on this hypothesis, this study is expected to generate novel insights into the pathophysiological mechanisms underlying the coexistence of DED and chronic pain, and to identify new therapeutic targets for both conditions.

In our study, we utilized GWAS data to explore the shared genetic etiology between DED and MCP. Initially, to understand the shared genetic basis of chronic pain and DED, we established genetic correlations. Subsequently, we employed cross-trait GWAS meta-analysis to identify common SNPs. Additionally, our study involved analyzing tissue-specific enrichment of genetic associations between chronic pain and DED. Finally, using SMR, we pinpointed genes with shared functionality between chronic pain and DED. Figure 1 presents a flowchart illustrating our analytical strategy.

**Figure 1:**
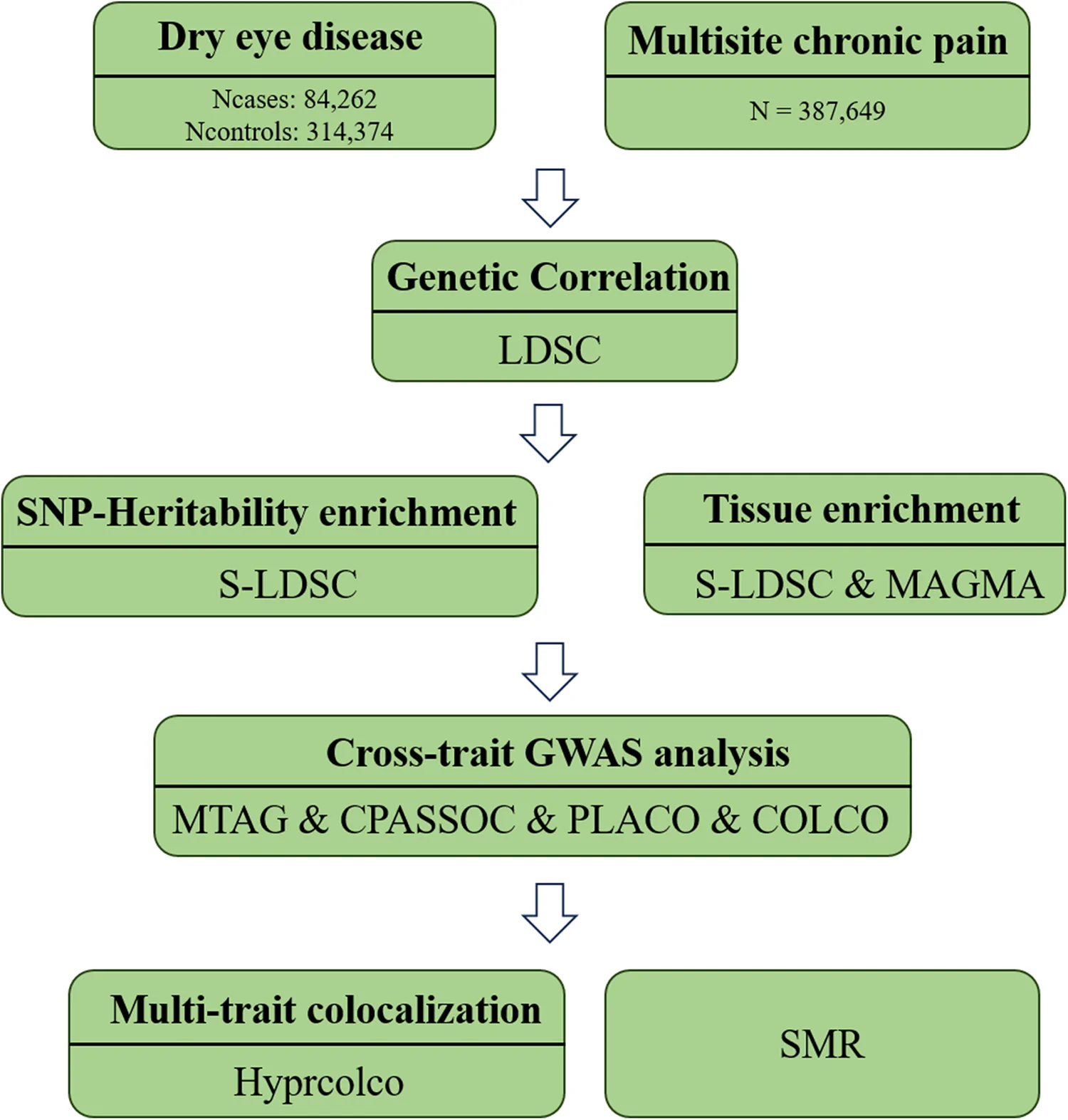
Flowchart of the study design.

## Methods

### Data sources

The GWAS data for multisite chronic pain (MCP) used in this study were derived from the study by Johnston et al., which was based on 387,649 European participants from the UK Biobank (13). MCP was defined as the number of chronic pain sites persisting for more than 3 months (ranging from 0 to 7 sites). The GWAS data for DED were obtained from the Million Veterans Program (14), based on PheCode Phe_375_1 (mapping to ICD-9-CM 375.15 and other diagnostic codes for dry eye syndrome) extracted from electronic health records, comprising 84,262 cases and 314,374 controls. Both studies were conducted in populations of European ancestry.

### Heritability and genetic correlation

Drawing upon GWAS summary-level data and European-lineage reference LD structures from the 1,000 Genomes Project, we conducted LDSC analysis (15) to characterize the heritable components of DED and MCP and to map their genetic overlap. The stratified extension of LDSC (16), coupled with the baseline LD annotation model (17), enabled heritability partitioning across a spectrum of continuous genomic features—departing from traditional binary annotation schemes. Bivariate LDSC with freely estimated intercepts served as our primary analytical framework for quantifying rg between DED and MCP; to account for putative sample duplication across GWAS sources, we repeated the analysis under an intercept-constrained model as a confirmatory sensitivity procedure.

### Partitioned heritability

To gain deeper insight into the genetic architecture shared between DED and MCP, we performed partitioned heritability analysis using S-LDSC (16). This approach classifies SNPs into biologically meaningful functional categories—such as coding regions, regulatory elements, and conserved sequences—and computes category-specific LD scores to quantify how each functional domain contributes to the overall SNP heritability of both traits. By estimating the proportion of heritability captured within each functional partition, we were able to dissect which genomic contexts disproportionately drive the genetic covariance between DED and MCP, thereby illuminating the molecular mechanisms underlying their shared polygenic basis.

### Tissue-specific enrichment of SNP heritability

We interrogated tissue-specific contributions to the DED-MCP genetic overlap using two convergent methods. S-LDSC (16) was first deployed with GTEx v8 reference panels (53 tissues) to test whether heritability enrichment clustered in specific anatomical contexts. It should be noted that GTEx v8 does not include ocular tissues, precluding direct assessment of eye-specific genetic regulation in this framework. We then corroborated these findings through MAGMA (18). The convergence of S-LDSC and MAGMA results allowed us to pinpoint the tissues most enriched for shared genetic signals between DED and MCP.

### Cross-trait GWAS meta-analysis

To identify risk SNPs jointly associated with both DED and MCP, this study conducted cross-trait meta-analyses using two methods: MTAG (19) and CPASSOC (20). In the MTAG analysis, by incorporating genetically correlated but phenotypically distinct traits, SNP effect estimates for each trait can be optimized. Furthermore, considering the differences in heritability between the two phenotypes, this study additionally employed CPASSOC for pairwise cross-trait meta-analysis. CPASSOC operates under the assumption of cross-trait heterogeneity in effects and calculates the cross-trait heterogeneity statistic SHet and its corresponding *P*-value through sample size-weighted meta-analysis. SHom, as a fixed-effect meta-analysis method, exhibits reduced statistical power in the presence of heterogeneity between studies; when effect sizes are heterogeneous, SHet serves as an extension of SHom and provides more robust statistical power. In this study, the SHet method was used to integrate summary statistics from DED and MCP.

The significance threshold for SNP selection was defined as a significant association with both phenotypes in both MTAG and CPASSOC analyses, with *P* < 5 × 10⁻⁸. After merging the summary statistics of DED and MCP using the SHet method, clumping analysis was performed using PLINK. Through the “clumping” function in PLINK, with parameters set as -clump-p1 5e-8 -clump-p2 1e-5 -clump-r2 0.1 -clump-kb 500, independent SNPs significantly associated with the phenotypes were identified.

### Genomic risk locus annotation and colocalization analysis

We used a three-step framework to identify and validate shared loci. Step 1 (Discovery): MTAG and CPASSOC were applied independently; genome-wide significant SNPs (*P* < 5 × 10⁻⁸) from each method were clumped (r^2^ < 0.1, 500 kb) to define independent candidate loci. Step 2 (Pleiotropy validation and locus definition): Independently, PLACO was applied to test for cross-trait pleiotropy, and FUMA was used to delineate genomic risk loci and define lead SNPs based on PLACO results. Step 3 (Colocalization and integration): COLOC was performed on all PLACO-defined loci to assess whether DED and MCP share the same causal variant (PP.H4 > 0.75). High-confidence shared risk loci were defined as those achieving genome-wide significance in both MTAG and CPASSOC (Step 1), residing within PLACO/FUMA-defined risk loci (Step 2), and exhibiting strong colocalization evidence (Step 3).

Based on the PLACO cross-trait association analysis results (21), FUMA was employed to perform genomic functional annotation and risk locus delineation for significant association signals (22). FUMA integrates GWAS summary statistics with reference panel linkage disequilibrium information to identify genomic risk loci and define independent significant SNPs and lead SNPs. The parameters were set as follows: lead SNP *P*-value threshold < 5 × 10⁻⁸, SNP inclusion *P*-value threshold < 0.05, independent significant SNPs defined at r^2^ ≥ 0.6 with the lead SNP, and lead SNPs required to be mutually independent at r^2^ < 0.1, with the reference panel selected from the 1000 Genomes Project Phase 3 European population. Additionally, FUMA was configured to perform eQTL mapping using the EyeGEx retinal eQTL dataset and 13 GTEx v8 brain tissues for all PLACO-defined risk loci.

To assess whether DED and MCP share the same causal variant at the risk locus level, Bayesian colocalization analysis was performed using COLOC (23). A posterior probability PP.H4 > 0.75 was considered as strong evidence for colocalization, suggesting that DED and MCP at that locus are highly likely to be driven by the same causal variant.

### Multi-trait colocalization

To evaluate the mediating role of brain imaging-derived phenotypes (IDPs) in the shared genetic mechanisms between DED and MCP, this study employed the HyprColoc analytical framework for multi-trait colocalization analysis (24). HyprColoc enables the simultaneous assessment of whether genetic association signals from multiple correlated traits share the same causal variant under a single causal variant assumption, thereby identifying potential mediating phenotypes or shared biological pathways.

The neuroimaging genetic data used in this study were derived from multimodal magnetic resonance imaging (MRI) findings of 33,224 European-ancestry participants from the UK Biobank (25). Following deduplication and reliability screening, 227 structural MRI (sMRI) phenotypes (including cortical thickness, cortical surface area, and subcortical volume) and 360 diffusion MRI (dMRI) phenotypes (including fractional anisotropy, mean diffusivity, and other white matter tract integrity indices) were retained for subsequent analysis (26).

First, based on the cross-trait significant loci for DED and MCP obtained from MTAG, CPASSOC, and PLACO, genetic association signals corresponding to the IDP regions were extracted. Subsequently, HyprColoc colocalization analysis was performed with DED, MCP, and candidate IDPs as input traits. If an IDP shared the same causal variant with DED and MCP (posterior cluster probability > 0.75), this would suggest that the brain imaging phenotype may participate in the common genetic pathway of both traits.

### Summary-data-based Mendelian randomization

SMR was employed to infer causal effects of gene expression on the phenotype (27), using genetic variants as instrumental variables. Significant associations were identified at P-SMR < 0.05 and subsequently retained only if P-HEIDI > 0.05 (28), ensuring the exclusion of linkage disequilibrium heterogeneity.

## Results

### Genetic correlation

LDSC genetic correlation analysis was performed using 1,169,034 SNPs after merging and QC. The unconstrained intercept model yielded a genetic correlation (rg) of 0.659 (SE = 0.032, *P* = 6.15 × 10^−92^). The LDSC intercepts were 1.032 (SE = 0.010) for MCP and 1.172 (SE = 0.008) for DED, with a genetic covariance intercept of 0.004 (SE = 0.006), suggesting minimal inflation from sample overlap or population stratification. The attenuation ratios were 0.054 for MCP and 0.461 for DED.

Given that the two GWAS were derived from non-overlapping cohorts (UK Biobank for MCP; Million Veterans Program for DED), we additionally performed a constrained-intercept sensitivity analysis (intercept fixed at 1 for heritability and 0 for genetic covariance), which yielded rg = 0.447 (SE = 0.015, *P* = 8.08 × 10^−185^) and SNP heritability estimates of 7.96% (SE = 0.002) for MCP and 5.37% (SE = 0.001) for DED. The consistency of significance across both models supports robust genetic correlation despite differences in absolute rg magnitude (Supplementary Table S1).

### Partitioned heritability analysis

SNPs partially genetically correlated with MCP exhibited enrichment in 40 out of 97 functional categories, with the leading category being Conserved_Primate_phastCons46wayL2_0 (enrichment = 10.8, *P* = 4.50 × 10⁻⁸). SNPs associated with DED showed enrichment in 19 out of 97 functional categories, also led by Conserved_Primate_phastCons46wayL2_0 (enrichment = 16.6, *P* = 0.003). A total of 11 functional categories demonstrated significant associations with both MCP and DED (Figure 2, Supplementary Table S2).

**Figure 2:**
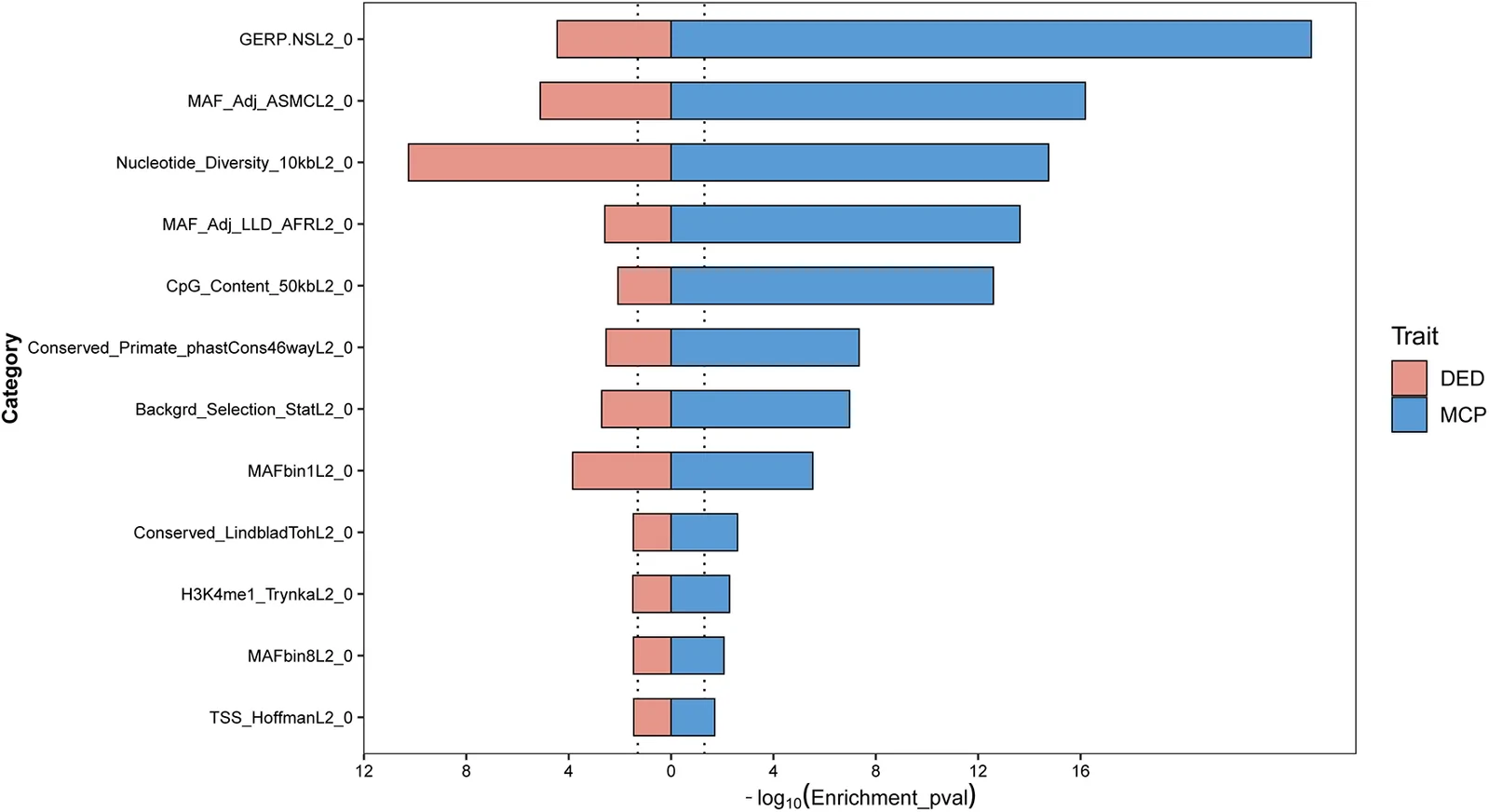
Partitioned genetic correlation between dry eye disease (DED) and multisite chronic pain (MCP).

### Tissue-level SNP heritability enrichment analysis

Using the S-LDSC method combined with multi-tissue gene expression data from the GTEx database, we evaluated the tissue-level SNP heritability enrichment characteristics of DED and MCP. The results revealed that both traits exhibited significant tissue-specific aggregation patterns of genetic signals, predominantly concentrated in nervous system-related tissues (Figure 3, Supplementary Table S3).

**Figure 3:**
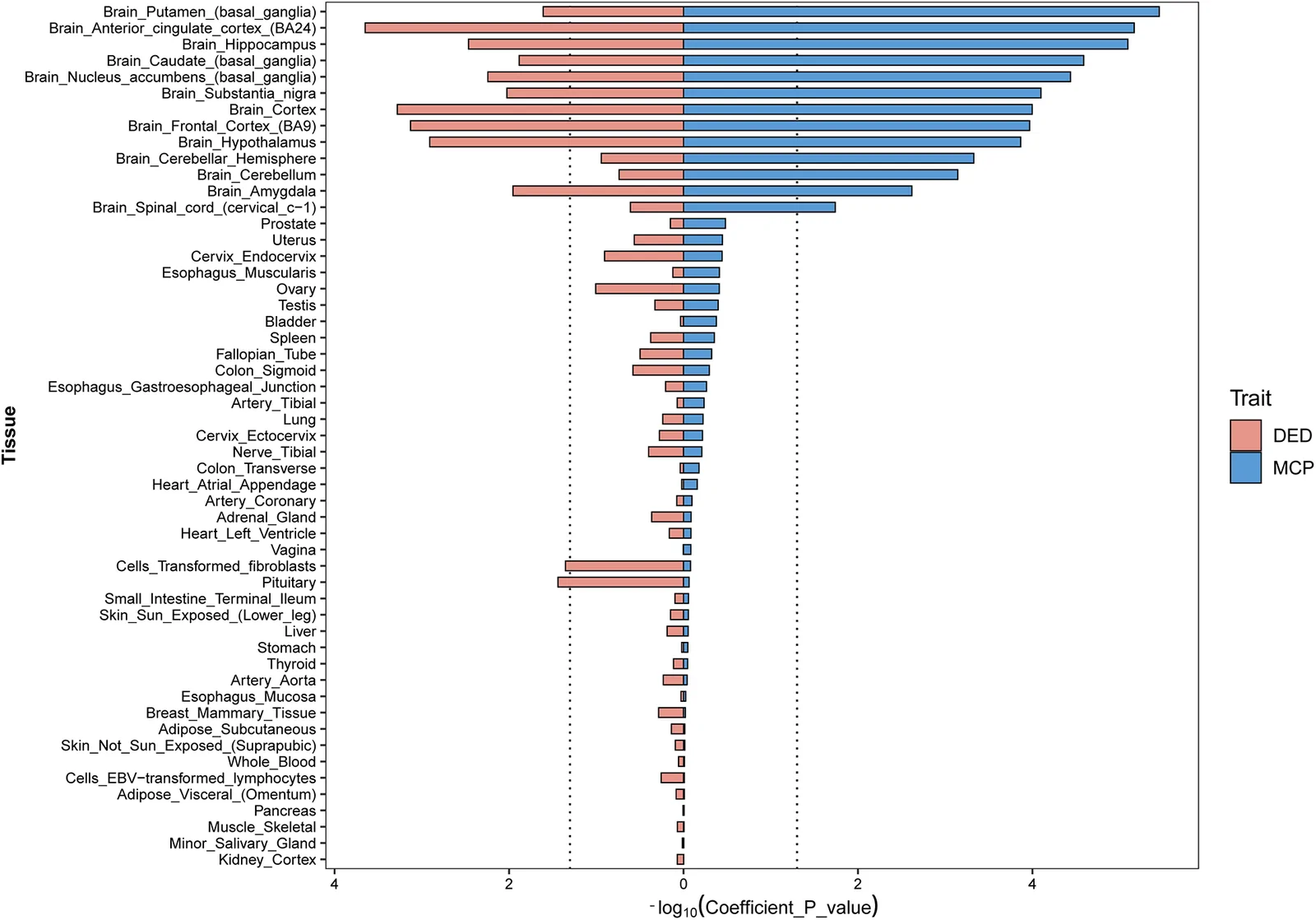
Tissue-specific enrichment of SNP heritability for dry eye disease (DED) and multisite chronic pain (MCP) by S-LDSC.

For DED, SNP heritability showed significant enrichment in 11 brain regions, with the anterior cingulate cortex (BA24), cerebral cortex, and frontal lobe demonstrating the most prominent enrichment. MCP SNP heritability exhibited significant enrichment in 14 brain regions, primarily in the putamen (basal ganglia), anterior cingulate cortex (BA24), and hippocampus. SNPs jointly associated with both traits displayed enrichment signals in 10 brain regions, with the anterior cingulate cortex (BA24) and amygdala as the core regions.

Furthermore, MAGMA tissue-specific analysis additionally revealed the tissue preferences of genetic regulation for DED and MCP. Risk SNPs for DED were specifically enriched in 2 brain regions, while risk SNPs for MCP showed enrichment in 11 brain regions. SNPs jointly associated with both traits were primarily enriched in the cerebellum and cerebellar hemispheres (Figure 4, Supplementary Table S4).

**Figure 4:**
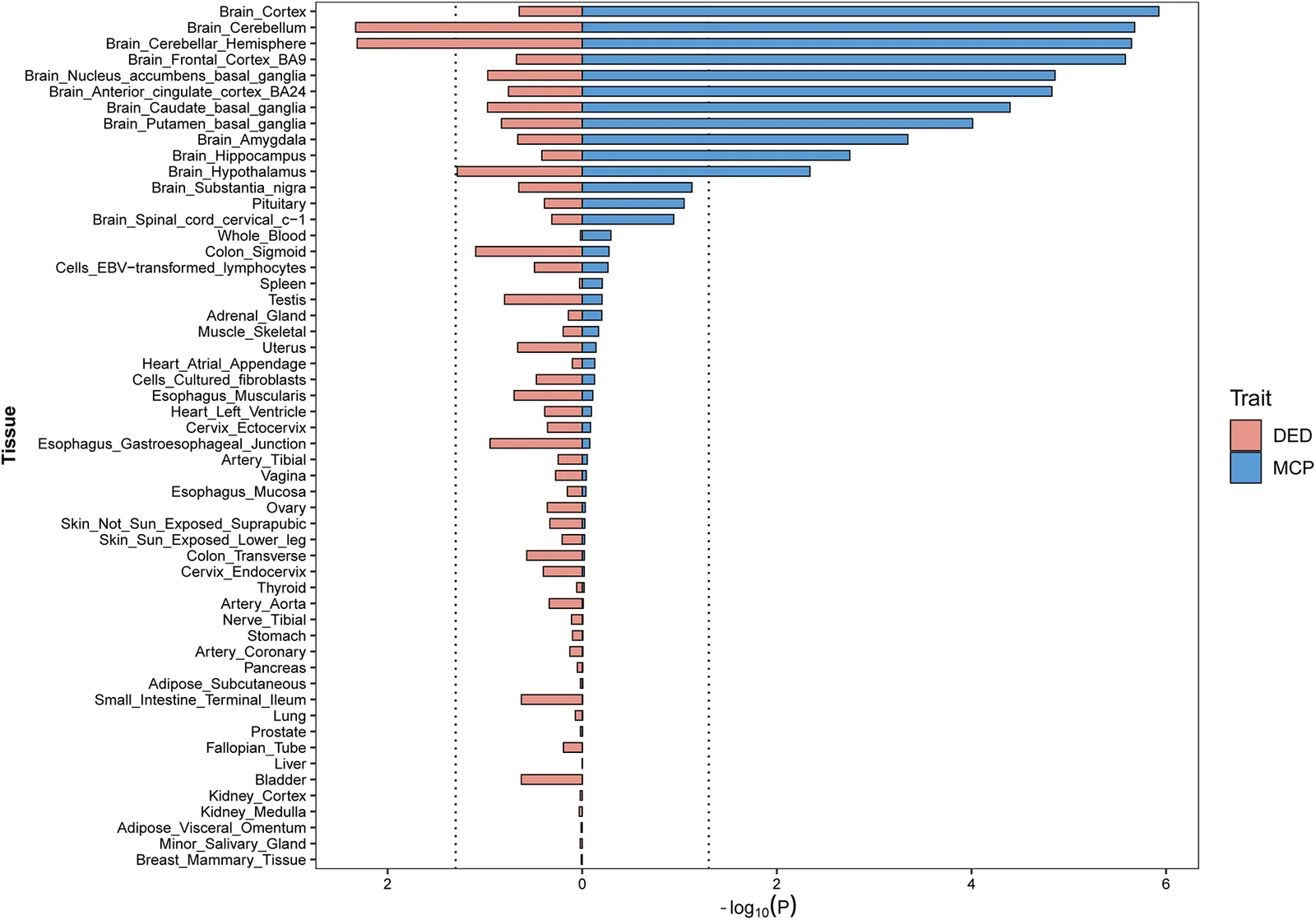
Tissue-specific enrichment of SNP heritability for dry eye disease (DED) and multisite chronic pain (MCP) by MAGMA.

### Cross-Trait GWAS meta-analysis and risk locus colocalization

To identify shared genetic loci, we first performed cross-trait meta-analyses using MTAG and CPASSOC, yielding 12 independent cross-trait significant SNPs. Notably, no SNP achieved genome-wide significance in both original DED and MCP GWAS simultaneously, confirming that these loci were discovered through cross-trait augmentation rather than simple overlap (Supplementary Table S5).

Independently, we applied PLACO to test for cross-trait pleiotropy and used FUMA to define genomic risk loci and lead SNPs based on the PLACO results, yielding 12 genomic risk loci (Figure 5). Bayesian colocalization (COLOC) was performed on all 12 PLACO-defined loci. Four loci exhibited strong evidence of shared causal variants (PP.H4 > 0.75; Supplementary Table S6). eQTL mapping of 12 PLACO-defined loci revealed that 16 of 43 mapped genes showed regulatory evidence (Supplementary Table S7). *SLC39A8* (rs13107325) exhibited significant retinal eQTL effects (EyeGEx; 60 SNPs, min *P* = 2.91 × 10^−10^). Brain-specific eQTLs were observed for 11 genes, with multi-tissue effects in *ZNF501* and *ZNF502* (9 regions including anterior cingulate cortex and hippocampus).

**Figure 5:**
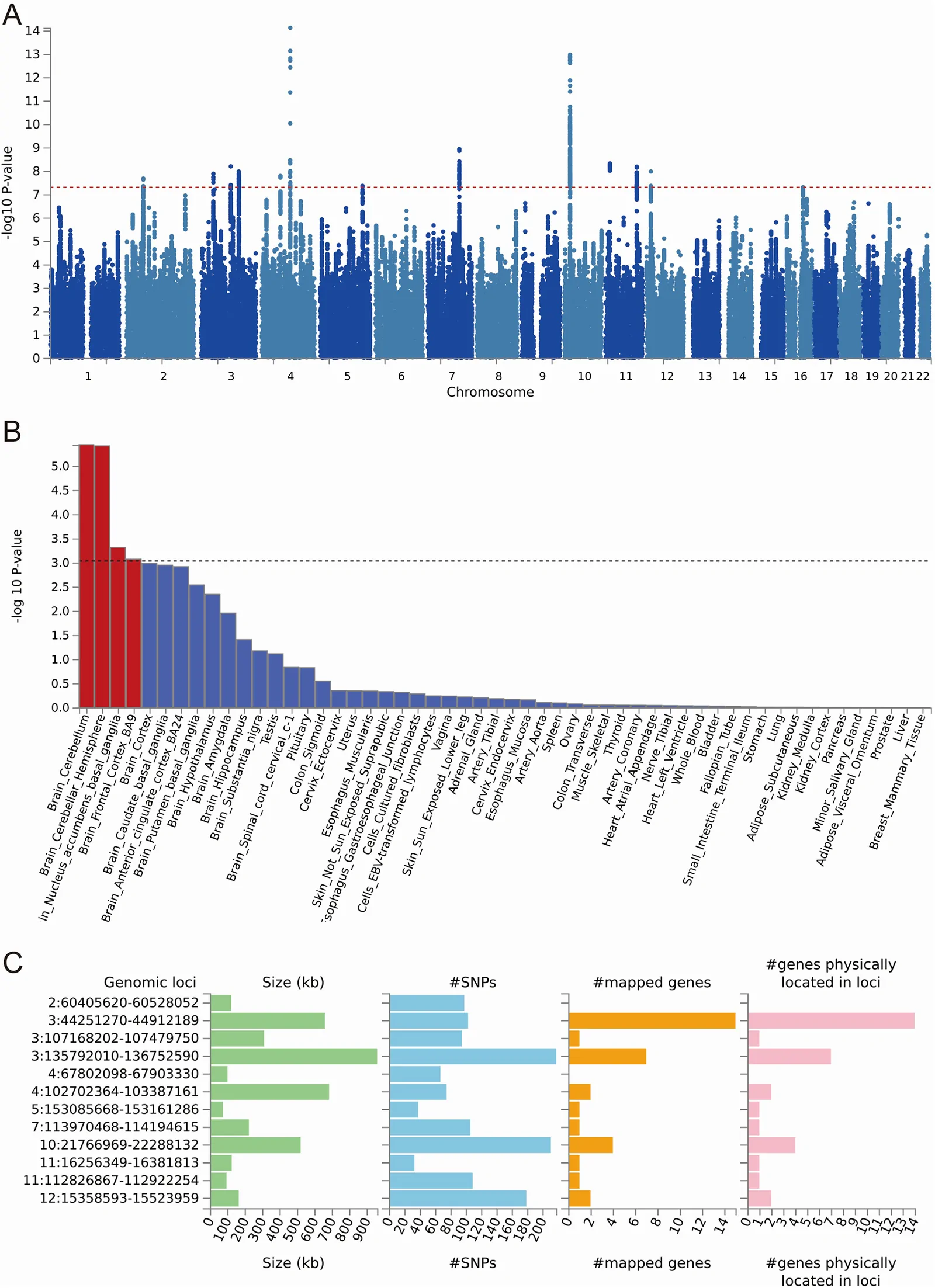
Functional mapping and annotation of shared genetic loci between dry eye disease (DED) and multisite chronic pain (MCP) identified by PLACO analysis. **(A)** Manhattan plot of PLACO meta-analysis results for DED and MCP. **(B)** Tissue-specific gene expression enrichment analysis based on GTEx v8 data. **(C)** Characteristics of the 12 independent genomic loci identified by FUMA.

We then integrated evidence across all three analytical stages. Three loci (rs13107325, rs7105462, rs7959961) met our stringent criteria for high-confidence shared risk loci, as they achieved genome-wide significance in both MTAG and CPASSOC (Step 1), served as lead SNPs within PLACO/FUMA-defined risk loci (Step 2), and showed strong colocalization (PP.H4 > 0.75; Step 3; Table 1). An additional PLACO locus demonstrated strong colocalization but was not genome-wide significant in both MTAG and CPASSOC (rs12253527). The remaining candidate SNPs from MTAG/CPASSOC that did not overlap with PLACO-defined risk loci, or lacked colocalization support, are considered candidate shared loci pending further validation.

**Table 1:** Shared risk regions in different analyses.

SNP	uniqID	Region	MTAG pval		CPASSOC pval	PLACO pval	PP.H4	Nearest genes
			MCP	DED				
rs13107325	4:103188709:C:T	4q24	8.30E-17	1.78E-15	4.42E-17	7.68E-15	0.9940789	SLC39A8
rs7105462	11:112912048:A:G	11q23.2	8.97E-10	2.64E-09	6.74E-10	6.70E-09	0.7756022	NCAM1
rs7959961	12:15379395:A:G	12p12.3	2.37E-08	6.10E-09	1.65E-09	1.06E-08	0.7582254	PTPRO

### Brain imaging phenotype mediation analysis

HyprColoc results demonstrated that the rs13107325 locus serves as a core hub for shared genetic signals between DED and MCP, showing significant three-way colocalization with 95 IDPs (posterior probability > 0.75). These IDPs were predominantly concentrated in the visual-parietal network and corticospinal motor pathways, encompassing bilateral lingual gyrus, cuneus, and lateral occipital cortical thickness, as well as neurite density index (ICVF) in the cerebral peduncle and anterior limb of the internal capsule. Additionally, bilateral cerebellar cortical volume also showed significant colocalization evidence (Supplementary Table S8).

### Identification of shared functional genes

SMR was applied to identify pleiotropic genes underlying both MCP and DED, integrating GWAS summary statistics for these traits with eQTL data from GTEx. S-LDSC and MAGMA analyses previously revealed convergent SNP heritability enrichment across the two conditions. Following HEIDI-outlier filtering, 21 genes were jointly implicated in MCP and DED (Figure 6, Supplementary Table S9).

**Figure 6:**
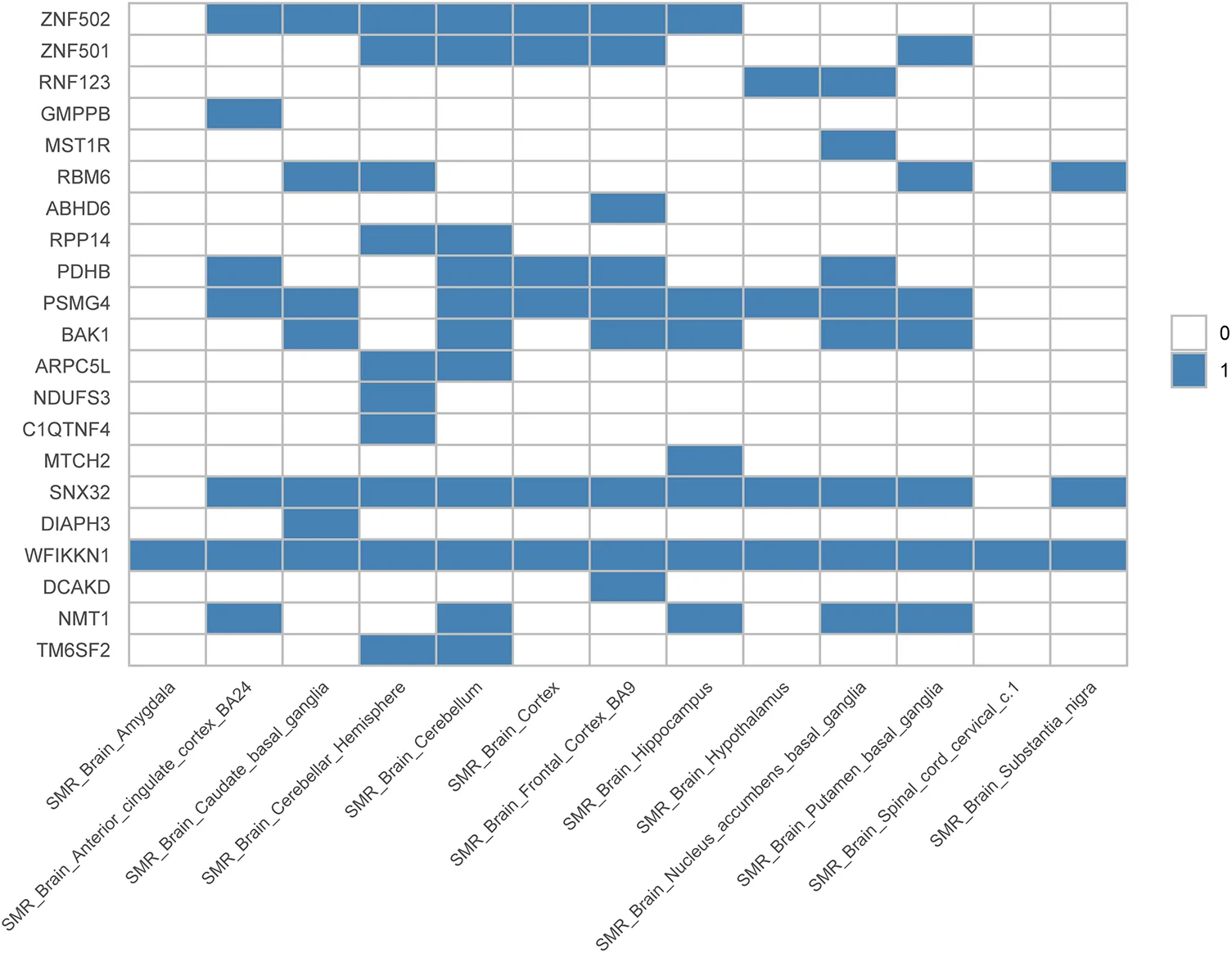
Overlap of significant genes associated with both DED and MCP in brain tissues identified by SMR.

## Discussion

To our knowledge, this study represents the first genome-wide cross-trait analysis to systematically evaluate the shared genetic basis between DED and MCP. Leveraging detailed GWAS datasets and tissue-specific expression data, our study revealed potential genetic links between DED and MCP. This is supported by several key observations: first, we identified specific genomic regions and functional elements associated with both DED and MCP. Second, our investigation of the genetic overlap between DED and MCP, particularly in brain tissues, led to the identification of numerous putative functional genes influencing both phenotypes.

A systematic review and meta-analysis revealed that, compared to healthy controls, patients with chronic pain exhibited more severe DED symptoms with significant clinical relevance; however, most patients showed minimal or even absent objective ocular surface signs (29). This “subjective symptoms outweighing objective signs” phenotype of DED suggests that both conditions share an underlying pathological basis of somatosensory hypersensitivity. It is currently widely accepted that the association between chronic pain and DED fundamentally involves dysfunctional nociceptive signal processing within the trigeminal somatosensory system, encompassing both peripheral and central sensitization mechanisms (30). Following disruption of somatosensory homeostasis and dysregulation of nociceptive pathways, neuropathic and nociplastic pain mechanisms are initiated (31, 32). Persistent activation of central sensitization can interfere with physiological tear film homeostasis and repair, resulting in sustained ocular pain in patients despite unremarkable findings on objective examination (30). Additionally, patients with fibromyalgia and irritable bowel syndrome commonly exhibit pathological alterations in corneal nerve morphology and reduced sub-basal corneal nerve fiber density (33, 34), further exacerbating dry eye.

To date, no study has systematically analyzed the genetic association between DED and MCP. Through multiple GWAS methods, this study identified three SNPs jointly associated with DED and MCP: rs13107325, rs7105462, and rs7959961, with neighboring genes *SLC39A8*, *NCAM1*, and *PTPRO*, respectively. Persistent nociceptive stimulation may alter *SLC39A8* expression, thereby triggering a cascade of responses including glial cell dysfunction, synaptic plasticity alterations, and structural reorganization in specific brain regions (35, 36). HyprColoc analysis further established rs13107325 as a critical genetic locus linking DED, MCP, and the central nervous system. *NCAM1* mediates peripheral nerve injury-induced synaptic plasticity reorganization in the anterior cingulate cortex, driving persistent sensitization of neuropathic pain (37). *PTPRO* is an essential receptor protein tyrosine phosphatase required for peptidergic differentiation and axonal targeting of dorsal root ganglion sensory neurons; its functional deficiency can lead to reduced nociceptive neuron numbers, abnormal spinal cord pathways, and impaired heat nociception (38).

Subsequent analysis of functional components identified 12 annotated regions associated with both DED and MCP. H3K4me1 demonstrated a significant negative correlation with pain severity, particularly in key pain-processing brain regions such as the periaqueductal gray, and may serve as a predictive marker for innate pain sensitivity (39). Backgrd_Selection_StatL2_0 and GERP.NSL2_0 have been demonstrated to be associated with migraine (40). These functional components related to DED require additional experimental validation.

Tissue enrichment analysis of key genes associated with DED and MCP revealed significant enrichment in brain tissues. S-LDSC analysis indicated that SNPs associated with both DED and MCP were enriched in 9 brain regions, predominantly concentrated in the anterior cingulate cortex. Similarly, MAGMA tissue-specific analysis demonstrated enrichment in 2 brain regions, particularly the cerebellum and cerebellar hemispheres. This enrichment, especially in the cerebellum and cerebellar hemispheres, suggests potential mechanisms underlying the coexistence of DED and MCP within brain structures. Compared with healthy individuals, DED patients exhibited significantly lower regional homogeneity values in the middle frontal gyrus, inferior frontal gyrus, and superior frontal gyrus (41), and demonstrated increased amplitude of low-frequency fluctuations (ALFF) in the bilateral supplementary motor area alongside decreased ALFF in the bilateral cerebellar lobules VIII/VIIb, left middle frontal gyrus, and right middle frontal gyrus on fMRI (42). The cerebellum, particularly the cerebellar hemispheres, plays a crucial role in the perception, processing, and modulation of chronic pain. Neuroimaging studies have confirmed that patients with chronic pain exhibit altered activation patterns and functional connectivity in the cerebellar hemispheres, involving interactions between the cerebellum and multiple brain regions including the limbic system, insula, and cortex (43, 44).

Through SMR, we explored functional genes associated with both conditions in specific enriched tissues, identifying 21 genes. Among these, *ZNF501* and *ZNF502* are located in the 3p21.31 neuropsychiatric hotspot region, are highly expressed in the dorsolateral prefrontal cortex, and are significantly associated with depression and anxiety risk (45); *ABHD6*, as an endocannabinoid 2-AG hydrolase, participates in pain threshold regulation and cortical spreading depression (46); *NDUFS3* is involved in mitochondrial energy metabolism (47). The roles of these identified genes in DED and MCP require further experimental validation.

Our study has several limitations. First, the GWAS data for both DED and chronic pain MCP were derived exclusively from European-ancestry populations, which may constrain the generalizability of our findings to non-European ethnic groups. Future investigations should therefore integrate multi-ethnic datasets to validate the trans-ancestry portability of the identified genetic signals. Second, the MCP summary statistics comprised a broad phenotypic spectrum with heterogeneous clinical definitions, potentially inflating sample size at the cost of etiological specificity. Subsequent analyses incorporating genetically informed subtyping—stratified by pain modality, anatomical distribution, or symptom chronicity—would offer a more granular understanding of how shared genetic architecture maps onto distinct clinical presentations. Third, our inferences are predicated solely on genetic variation, whereas epigenetic modifications and gene-environment interplay constitute equally important, yet unexamined, layers of biological complexity underlying DED-MCP comorbidity. Fourth, GTEx v8 lacks ocular tissues, precluding eye-specific enrichment assessment in S-LDSC and MAGMA. Although ocular eQTL resources (e.g., EyeGEx) are available, they are not compatible with these standard enrichment frameworks. Future integration of eye-specific transcriptomic data remains an important direction.

## Conclusion

In summary, this study identified significant genetic overlap between DED and MCP, with three pleiotropic risk variants (rs13107325, rs7105462, and rs7959961) further corroborating this finding. The shared genetic architecture exhibited pronounced brain region specificity. Integrating transcriptomic data, we identified 21 candidate functional genes. These findings elucidate the polygenic pathogenic basis of DED comorbid with chronic pain, refine the neurobiological theory linking ocular surface dysfunction with central pain modulation, and provide a theoretical foundation for developing targeted therapeutics targeting common molecular pathways and personalized treatment strategies that simultaneously improve both conditions.

## Data Availability

The original contributions presented in the study are included in the article/Supplementary Material, further inquiries can be directed to the corresponding author.
